# Large-scale clustering of CAGE tag expression data

**DOI:** 10.1186/1471-2105-8-161

**Published:** 2007-05-21

**Authors:** Kazuro Shimokawa, Yuko Okamura-Oho, Takio Kurita, Martin C Frith, Jun Kawai, Piero Carninci, Yoshihide Hayashizaki

**Affiliations:** 1 Genome Exploration Research Group, RIKEN Genomic Sciences Center (GSC), RIKEN Yokohama Institute, 1-7-22 Suehiro-cho, Tsurumi-ku, Yokohama, Kanagawa 230-0045, Japan; 2 National Institute of Advanced Industrial Science and Technology, Tsukuba, Ibaraki 305-8568, Japan; 3 Institute for Molecular Bioscience, University of Queensland, Brisbane, Qld 4072, Australia; 4 Genome Science Laboratory, Discovery Research Institute, RIKEN Wako Institute, 2-1 Hirosawa, Wako, Saitama 351-0198, Japan

## Abstract

**Background:**

Recent analyses have suggested that many genes possess multiple transcription start sites (TSSs) that are differentially utilized in different tissues and cell lines. We have identified a huge number of TSSs mapped onto the mouse genome using the cap analysis of gene expression (CAGE) method. The standard hierarchical clustering algorithm, which gives us easily understandable graphical tree images, has difficulties in processing such huge amounts of TSS data and a better method to calculate and display the results is needed.

**Results:**

We use a combination of hierarchical and non-hierarchical clustering to cluster expression profiles of TSSs based on a large amount of CAGE data to profit from the best of both methods. We processed the genome-wide expression data, including 159,075 TSSs derived from 127 RNA samples of various organs of mouse, and succeeded in categorizing them into 70–100 clusters. The clusters exhibited intriguing biological features: a cluster supergroup with a ubiquitous expression profile, tissue-specific patterns, a distinct distribution of non-coding RNA and functional TSS groups.

**Conclusion:**

Our approach succeeded in greatly reducing the calculation cost, and is an appropriate solution for analyzing large-scale TSS usage data.

## Background

Large amounts of gene expression data are now available, generated by the well-known oligonucleotide chip, cDNA microarray, and serial analysis of gene expression (SAGE) techniques, as well as by new tiling array techniques [1-5]. These techniques are used in large-scale gene expression analyses for classifying gene expression patterns [6,7]. However, most of the techniques can only recognize a group of transcript variants as a single transcript, because all variants hybridize to the same probe on the arrays, hiding the distinct expression regulation of each variant reflecting the condition of tissues and developmental stages.

Recently, we have developed a gene expression measurement technique, called cap analysis of gene expression (CAGE), which effectively detects distinct transcription start site sequences, and marks the location with what we call CAGE tags. CAGE tags are grouped into tag clusters (TCs), where the member tags map to the same strand of a chromosome and overlap by at least 1 bp. Analysis of TCs enables us to recognize representative TSSs and their upstream regulatory elements [8]. Massive CAGE analysis in the Functional Annotation of Mouse (FANTOM) 3 activity has shown that one gene locus, or transcription unit (TU), can have as many as three TCs on average, resulting in alternative transcripts [9]. Some of these alternative transcripts are translated into proteins with distinct biological functions [10,11]. Therefore, identifying TSSs is an essential process for researching the mechanisms that regulate gene expression in a variety of tissues and developmental stages. It is also important to quantify the absolute expression values for each TSS, rather than the relative expression level compared to reference RNA. In this context, CAGE analysis indicates discrete TSSs expression intensities. Using these characteristic features of CAGE analysis, we have developed a calibration method to exchange relative expression values for absolute counts of mRNA in a sample [12].

As a consequence of the complex features of transcriptional gene expression regulation, the number of TCs (equal to representative TSSs) that needs to be analyzed totals 159,075 (from 127 mouse samples) from FANTOM3 [9], far exceeding the number of actual genes [13]. If we were to attempt analyzing this CAGE data, the large number of TSSs might prevent a realistic determination of the solution due to the sheer size. Therefore, more effective systems need to be developed for processing the expected amount of TSS data. Although various methods have been reported for clustering of expression data [14], some of them are difficult to use, having high computational requirements. For instance, it is difficult for a standard 32 bit personal computer to process more than 40,000 genes using hierarchical clustering, for the algorithm consumes more than 8G bytes of memory.

Here, we report the system and methods of a two-step clustering of CAGE TSS data in detail, where we combine different clustering methods. By using non-hierarchical clustering, we can save computational power, even if the amount of data surpasses the ordinarily computable amount of data for hierarchical clustering. The Usage of this two-step method in FANTOM3 activity has been already succeeded in showing a part of the clustering results such as the relations between different clusters and the classification of a huge amount of upstream sequence of the CAGE tags [9]. Then, here we will compare two results of the clustering in different numbers of clusters, and assess the validity of our method by examining whether clustering data indicates molecular functions annotated by GO terminology. Both results seem to provide the visually understandable hierarchical tree structure that can be widely used by biologists. This attempt, here we would demonstrate, let us confirm cluster data agreed with past biological findings reflected in GO terms, and notice new findings about aging-related genes. Besides the systemic determination of gene network architecture in yeast [15], our approach is the first to cluster TSS groups for whole-genome transcripts, including non-coding RNA.

## Systems and Methods

### Overview of clustering strategy

The clustering method has two steps. The first step is a robust process to divide the dataset into small enough parts to enable the employment of hierarchical clustering algorithms. The division is performed with the k-means method, which is suitable for large amounts of gene expression data littered with noise [16,17]. This needs a lower calculation order (O (*Nk*); *N*: number of data items; *k*: number of clusters) and demands less computational memory than the hierarchical method (which needs O (*N*^2^) of memory and more than O (*N*^2^) of the corresponding calculation order).

**Figure 1:**
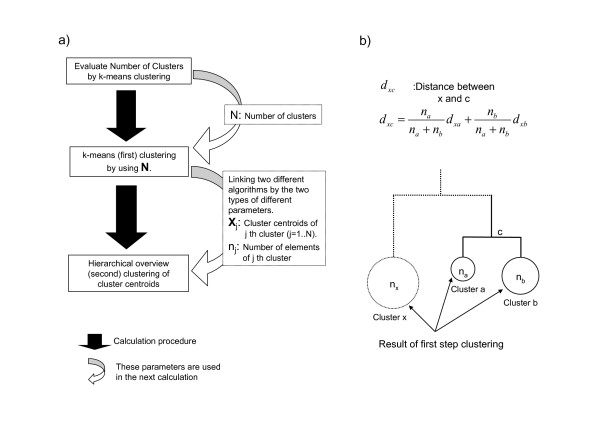
**Calculation procedure of the two step clustering**. a) Schematic diagram of our clustering method. The second step needs two types of parameters, cluster centroid vector and number of elements of each cluster, calculated in the first step. b) Detail of the link algorithm. Each cluster calculated in the first step is connected by our method. This figure is an example of the use of the average-linkage algorithm for the second step clustering.

The second step of the analysis is done with hierarchical clustering (for an overview of tree clustering and hierarchical clustering of individual groups, see Figure 1), based on the calculation results from the first step, such as the cluster centroid and number of TSSs in each cluster. This two-step clustering method provides us with a good graphical representation reflecting the biological significance of the FANTOM3 data, which is easy to analyze statistically and gives a biologist a general view of the data in order to analyze clusters. Figure 1a,b shows our calculation procedure.

### Number of clusters

Non-hierarchical methods (similar to k-means) require a suitable number of clusters. However, it is difficult to decide the optimal number of clusters and the question of how many clusters to use has been discussed and there are several ways to decide the cluster number [15,18,19]. To decide the optimum number of groups, information criteria such as Akaike's Information Criterion (AIC) and Minimum Description Length (MDL) [20,21] or simple functions such as the mean square error (MSE) can be used. In our case, we have evaluated this number by using the normalized residual sum of squares (nRSS), which is based on the statistical characteristics of the data. The nRSS is one of the simpler prediction functions given by the following equation, to estimate and validate the number of clusters:

nRSS=∑l=1L1nl(∑i=1nl|x→l,i−x→l|2)
 MathType@MTEF@5@5@+=feaafiart1ev1aaatCvAUfKttLearuWrP9MDH5MBPbIqV92AaeXatLxBI9gBaebbnrfifHhDYfgasaacH8akY=wiFfYdH8Gipec8Eeeu0xXdbba9frFj0=OqFfea0dXdd9vqai=hGuQ8kuc9pgc9s8qqaq=dirpe0xb9q8qiLsFr0=vr0=vr0dc8meaabaqaciaacaGaaeqabaqabeGadaaakeaaieaacqWFUbGBcqWFsbGucqWFtbWucqWFtbWucqGH9aqpdaaeWbqaamaalaaabaGaeGymaedabaGaemOBa42aaSbaaSqaaiabdYgaSbqabaaaaaqaaiabdYgaSjabg2da9iabigdaXaqaaiabdYeambqdcqGHris5aOWaaeWaaeaadaaeWbqaamaaemaabaGafmiEaGNbaSaadaWgaaWcbaGaemiBaWMaeiilaWIaemyAaKgabeaakiabgkHiTiqbdIha4zaalaWaaSbaaSqaaiabdYgaSbqabaaakiaawEa7caGLiWoadaahaaWcbeqaaiabikdaYaaaaeaacqWGPbqAcqGH9aqpcqaIXaqmaeaacqWGUbGBdaWgaaadbaGaemiBaWgabeaaa0GaeyyeIuoaaOGaayjkaiaawMcaaaaa@5501@

where the cluster number is *l *{*l *= 1,..., *L*} where L is the total number of clusters, the number of TCs in each cluster is *n*_*l *_(*l *= 1,..., *L*), and TC expression vectors in each cluster *l *are x→
 MathType@MTEF@5@5@+=feaafiart1ev1aaatCvAUfKttLearuWrP9MDH5MBPbIqV92AaeXatLxBI9gBaebbnrfifHhDYfgasaacH8akY=wiFfYdH8Gipec8Eeeu0xXdbba9frFj0=OqFfea0dXdd9vqai=hGuQ8kuc9pgc9s8qqaq=dirpe0xb9q8qiLsFr0=vr0=vr0dc8meaabaqaciaacaGaaeqabaqabeGadaaakeaacuWG4baEgaWcaaaa@2E37@_*l*,*i *_{*i *= 1,..., *n*_*l*_}. The cluster centroid vector of x→
 MathType@MTEF@5@5@+=feaafiart1ev1aaatCvAUfKttLearuWrP9MDH5MBPbIqV92AaeXatLxBI9gBaebbnrfifHhDYfgasaacH8akY=wiFfYdH8Gipec8Eeeu0xXdbba9frFj0=OqFfea0dXdd9vqai=hGuQ8kuc9pgc9s8qqaq=dirpe0xb9q8qiLsFr0=vr0=vr0dc8meaabaqaciaacaGaaeqabaqabeGadaaakeaacuWG4baEgaWcaaaa@2E37@_*l*, *i *_{*i *= 1,..., *n*_*l*_} is x→¯
 MathType@MTEF@5@5@+=feaafiart1ev1aaatCvAUfKttLearuWrP9MDH5MBPbIqV92AaeXatLxBI9gBaebbnrfifHhDYfgasaacH8akY=wiFfYdH8Gipec8Eeeu0xXdbba9frFj0=OqFfea0dXdd9vqai=hGuQ8kuc9pgc9s8qqaq=dirpe0xb9q8qiLsFr0=vr0=vr0dc8meaabaqaciaacaGaaeqabaqabeGadaaakeaacuWG4baEgaWcgaqeaaaa@2E4E@_*l*_. To validate this model, we used the 10-fold cross validation method. All of the CAGE TC data were randomly divided into 10 sub-groups. Dataset *D*(- *j*) was a combination of 9 sub-groups other than *j *(1 ≤ *j *≤ 10), and was used for estimation of the number of clusters. The index for estimation, nRSS(e), was the average of the results from the calculation by the equation when *j *was changed from 1 to 10 in dataset *D*(- *j*). Dataset *D*(*j*) was the *j *th sub-group, which was used to validate the estimated result. The index for validation, nRSS(v), is the calculated result using dataset *D*(*j*).

### Link algorithm

To connect the non-hierarchical (first) clustering result with the hierarchical (second) clustering algorithms, and to draw a clustering overview tree, we used the information of cluster centroids and the number of cluster members from the first step. The application of these non-hierarchical clustering results is different depending on the link algorithm used in the hierarchical clustering. Here, we mention two cases. In the case of average-linkage algorithms, the equation of the link algorithm is as follows:

dxc=nana+nbdxa+nbna+nbdxb
 MathType@MTEF@5@5@+=feaafiart1ev1aaatCvAUfKttLearuWrP9MDH5MBPbIqV92AaeXatLxBI9gBaebbnrfifHhDYfgasaacH8akY=wiFfYdH8Gipec8Eeeu0xXdbba9frFj0=OqFfea0dXdd9vqai=hGuQ8kuc9pgc9s8qqaq=dirpe0xb9q8qiLsFr0=vr0=vr0dc8meaabaqaciaacaGaaeqabaqabeGadaaakeaacqWGKbazdaWgaaWcbaGaemiEaGNaem4yamgabeaakiabg2da9maalaaabaGaemOBa42aaSbaaSqaaiabdggaHbqabaaakeaacqWGUbGBdaWgaaWcbaGaemyyaegabeaakiabgUcaRiabd6gaUnaaBaaaleaacqWGIbGyaeqaaaaakiabdsgaKnaaBaaaleaacqWG4baEcqWGHbqyaeqaaOGaey4kaSYaaSaaaeaacqWGUbGBdaWgaaWcbaGaemOyaigabeaaaOqaaiabd6gaUnaaBaaaleaacqWGHbqyaeqaaOGaey4kaSIaemOBa42aaSbaaSqaaiabdkgaIbqabaaaaOGaemizaq2aaSbaaSqaaiabdIha4jabdkgaIbqabaaaaa@4EBF@

where *a*, *b*, *c*, *x *is the specific cluster number, *d*_*xa *_is the distance between cluster *a *and cluster *x *and *n*_*a *_is the number of members in cluster *a*. Note that, at the first merging, *n*_*a*_, *n*_*b*_,....*n*_*x *_are the number of the members in each cluster, which is calculated by k-means clustering (see Figure 1b). In the original average-linkage algorithm, this value is always 1. We substituted these values with the result of the non-hierarchical clustering (the first step). This equation will depend on the hierarchical clustering link algorithm chosen by the user. In the case of complete linkage algorithms, our method does not affect the calculation because the algorithm does not use the number of cluster members.

## Results

### Decision of number of clusters

**Figure 2:**
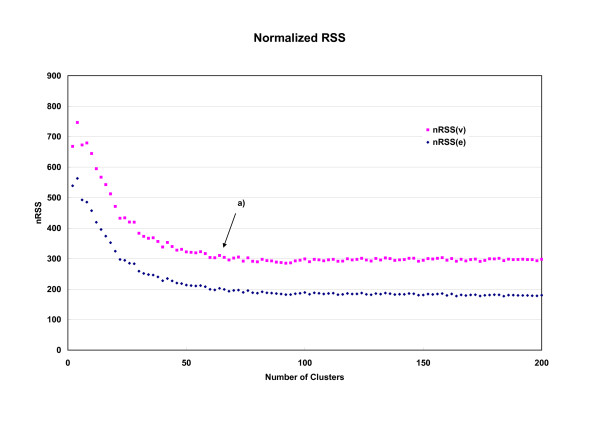
**Scatter chart of averaged nRSS index versus number of clusters**. The data set is the CAGE tag cluster expression data set from FANTOM3. The number of clusters was estimated by the 10-fold cross validation method. nRSS(e): The Normalized residual sum of squares results calculated by using the estimation dataset. nRSS(v): Result of the validation dataset: a): The value of the number of clusters at which the nRSS(v) reaches the minimum.

Figure 2 shows the relationship between number of clusters and values of nRSS indices, nRSS(e) and nRSS(v), calculated with the estimation dataset and the validation dataset, respectively (see Systems and Methods). The value of nRSS decreased as the number of clusters increased, as is expected from the inverse relation of these two parameters; thus, a local minimum of the value of nRSS(v) can be settled in the selected range (0 to 200) of the number of clusters. Indeed, in an approximation with the nRSS index, the index value approached a certain constant value when the number of clusters reached 70. From this index, we decided that the optimum number of clusters is 70 (*L *= 70 in equation (1)). Moreover, by substituting this number with 100 in the following example below, we were able to show that the change has no essential influence on the analytical result, giving no reason for further division of the CAGE data. In the second step clustering, we applied the hierarchical average-linkage algorithm to the 70 and 100 cluster groups. The most important point in this step is that the number of elements in the hierarchical clustering calculation decreases from 159 075 (TSSs) to 70 or 100 (clusters). The size of memory needed to calculate the Cophenetic matrix, essential for the processing of this clustering algorithm, decreases drastically from 126G bytes to 24K bytes. As a result, hierarchical clustering algorithms can be executed even when the number of data points is huge. In the next section, we focus on the clustering result divided into 70 clusters, and point out that the result agrees with existing findings. Afterwards, we discuss the resulting differences between the calculation of data divided into 70 or 100 clusters.

### Verification of two step clustering

#### 1. Expression pattern

**Figure 3:**
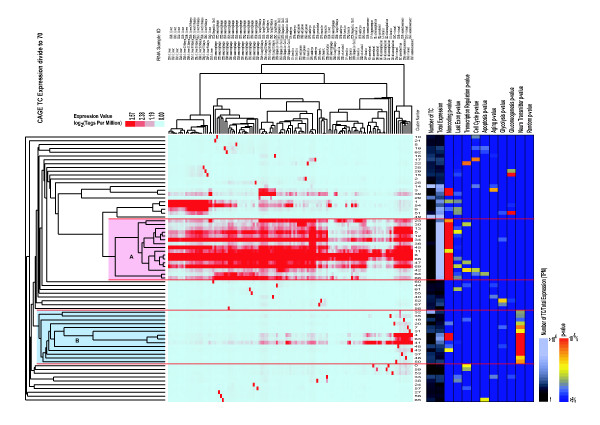
**Tree view image and supergroup annotation for 70 clusters**. The tree view of the hierarchical clustering of CAGE expression data (left side of figure), and the number of tag clusters (TCs, shaded) and *P*-values (color-coded) of TCs included in each cluster classified by GO terms (right side of figure). This figure displays the results of 159 075 TCs from 127 RNA samples. Hierarchical clustering was performed for 70 clusters which were grouped by the k-means method, and for 127 RNA samples.

Figure 3 shows the clustering results of CAGE expression data (divided into 70 clusters). Among these clusters, several supergroups, cluster families with distinct expression patterns and biological features, appeared. Supergroup A_(70)_,(A _(70)_: group A is part of the dataset which was divided into 70), which is composed of clusters 23_(70) _to 42_(70) _(not numerical order; see Figure 3, formed a large "ubiquitous and high expression" group, including TCs expressed in most examined mouse organs, tissues, and cell lines. This group was characterized by a lower "number of TCs", which means that the clusters are composed of a small number of transcripts each (710 per cluster on average), and that they have a larger "total expression": the total amount of expression per cluster was more than 10 000 tags per million (except cluster 23_(70)_).

Clusters outside supergroup A_(70)_, formed several other supergroups, characterized by tissue-specific expression patterns. The clusters 3_(70)_, 4_(70)_, 39_(70)_, 41_(70)_, 63_(70)_, and 64_(70) _that did not form supergroups, were characterized by broad and high expression levels with tissue-specific expression patterns. Clusters 3_(70) _and 39_(70) _were dominantly expressed in the lung, cluster 64_(70) _in macrophages, and clusters 4_(70)_, 41_(70) _and 63_(70) _in the brain. Supergroup A and these 7 clusters form a "broad and high expression" cluster family. In contrast to this, clusters 49_(70) _and 14_(70) _were "broad and low expression" clusters, enclosing the largest and the second-largest numbers of TCs (38 078 and 13 786 per cluster, respectively), and therefore resulting in low expression values (5 to 6 tags per million on average), even if the total expression in each cluster was high.

**Figure 4:**
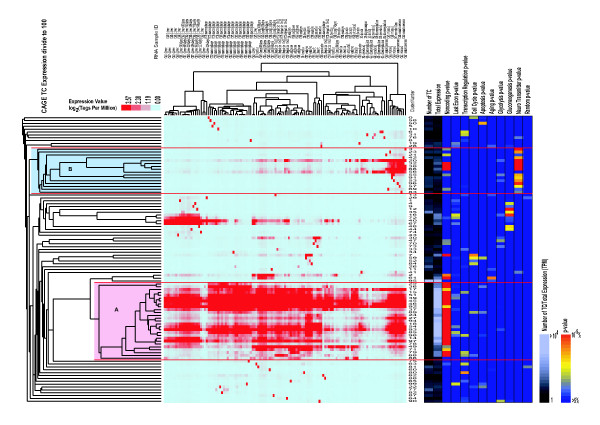
**Tree view image and supergroup annotation for 100 clusters**. Hierarchical clustering result, performed for 100 clusters which were grouped by the k-means method.

**Figure 5:**
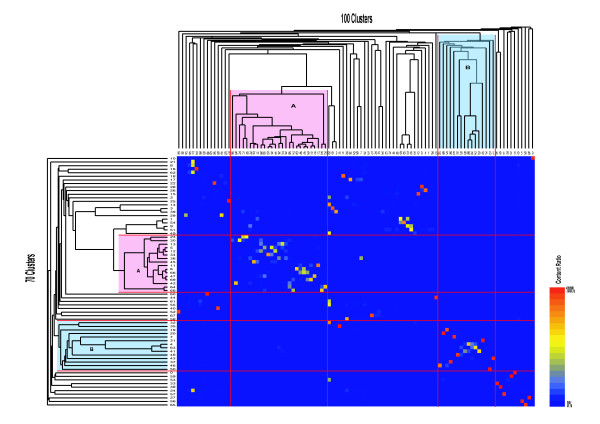
**The splitting and merging of the TCs**. The splitting and merging of the TCs between the two clusterings (content ratio), generating 70 (Fig. 3) and 100 (Fig. 4) distinct clusters, are shown here. The expression level for each cluster is the value of the cluster centroid; "Numbers of TCs" shows the number of tag clusters in each cluster. "Total Expression" shows the total expression level (log_2_(tags per million)) in each cluster. "Random" shows the result analyzed by using 5,000 TCs of different GO terms, chosen at random. "*P*-value" shows the statistical probability of the accuracy of the tag clusters classified by gene ontology terms. See Systems and Methods for how tag clusters are classified by gene ontology.

To evaluate the difference in the result that may be caused by different numbers of clusters, we compared the relation of the supergroups and clusters in two datasets where the data were divided into either 70 or 100 clusters. Figure 4 is a result of the hierarchical clustering (the second step) using the data divided into 100 clusters, and there are no significant differences between the two cluster numbers in Figure 3 and 4 when we compare it with our earlier results. Figure 4 has the supergroups A, B and tissue-specific clusters similar to the structures observed in Figure 3. Figure 5 shows the number of TC overlaps between the two data sets. About 91% of the TCs belonging to supergroup A_(100)_, belong also to supergroup A_(70)_. The lung specific cluster 61_(100)_, consists in part of cluster 3_(70)_, and cluster 2_(100) _is partly made up by cluster 39_(70)_. Clusters 66_(100)_, 20_(100) _and 32_(100)_, which are mostly expressed in the brain, correspond to the clusters 4_(70)_, 41_(70)_, and 63_(70)_, respectively, further decreasing the difference between the two data sets.

#### 2. Gene ontology terms

To validate the biological relevance of the clusters, we identified cluster-specific gene ontology (GO) terms by *P*-values [22].

The ontologies are structured as directed acyclic graphs, which are similar to hierarchies. A more specialized term has parents of a more generalized term. If a general term is selected, it may contain several GO terms with opposite biological meanings: for example, a general GO term, apoptosis (GO:0006915), contains anti-apoptosis (GO:0006916) and induction of apoptosis (GO:0006917). Then, we first selected specialized GOs related to organ-specific functions: glycolysis, gluconeogenesis, and neurotransmitters [23], to test whether the transcripts in the clusters with tissue-specific expression patterns could be annotated by proper GO terms. Indeed, TCs in supergroup B_(70)_, which is expressed in the brain, were tightly related to the GO category for neurotransmitters; TCs in cluster 52_(70)_, which originated from muscle tissues, were annotated for glycolysis; and TCs in cluster 51_(70) _and 16_(100)_, dominantly expressed in the liver, were characterized by GOs for gluconeogenesis (Figure 3, 4). The consistency between the GO terms and the expression patterns in the clusters allowed us to propose the function of transcripts as involved in the distinct biological activities of these organs.

Because transcripts have a wide variety of biological functions, our samples, mostly derived from normal mice, were not diverse enough to provide appropriate TCs for all GOs, but they were enough to elicit the TCs obtained in particular conditions and to form concordant clusters with more general GO terms. TCs in cluster 17_(70) _was largely derived from embryos (CFT to CFW; Figure 3) and was annotated by GOs for cell cycle, which is reasonable given the active cell division found in embryos. TCs in cluster 65_(70)_, with a prominent *P*-value for apoptosis (10^-5^), originated from macrophages, which undergo apoptosis [9].

We compared the members of TCs formed by two different numbers of clusters (70 and 100 clusters in figure 3 and 4, respectively). The correspondence of the clusters formed in the two different clusterings is shown in figure 5. Most of the TCs in supergroup A_(70) _and B_(70) _belong to corresponding clusters in supergroup A_(100) _and B_(100) _respectively Further, we were able to show their prominent *P*-values of GOs in the clusters, showing similar GO p-values to those of the corresponding clusters. For example, the clusters 50_(100) _and 90_(100) _contained almost the same TCs as 65_(70) _and 52_(70)_, respectively resulting in mostly the same GO *P*-values. Because the TC members and their GO *P*-values of the clusters was thereby almost the same, regardless of the cluster number, we could assume that our attempts to annotate the clusters by GO succeeded well, and the results were not influenced by differences in the number of clusters.

#### 3. Non-coding RNA

In the FANTOM3 activity we cloned 34 030 non-coding transcripts, which comprised 33% of the total transcripts [9]. We tested whether there was any tendency of non-coding TC expression (non-coding *P*-value in Figure 3, 4). A large number of non-coding TCs (1,318 non-coding TCs in 11,264 TCs) were contained in the "broad and high expression" supergroup A_(70) _(*P*-values < 10^-6 ^except clusters 11_(70)_), but not in the "broad and low expression" clusters (*P*-values > 5%) or "other tissue-specific" clusters (*P*-value > 10^-3^). Thus, in this computational method, the number of non-coding TCs detected with this CAGE analysis is tightly related to the proportion of TCs with broad and high expression levels. In the data set divided into 100, the results are similar: the majority of the non-coding TCs appear in supergroup A_(100) _(1,348 non-coding TCs in 11,521 TCs) with "broad and high expression". Thus, our method is more suitable to analyze statistical tendencies across several clusters than the k-means method alone, since k-means cannot describe or detect the relations between clusters.

## Discussion

Genome-wide surveys of gene expression are gaining in importance, and computational methods capable of handling the enormous amount of information generated are sorely needed. Here we propose a mathematical clustering method using genome-wide TSS data derived from transcripts in various organs, tissues, and cell lines of mice from the FANTOM3 consortium [9]. The study of sequence-based TSSs has unique problems. One problem is the difficulty in dealing with the entire data set, because the amount of data is huge and much larger than the number of genes.

To solve this problem, we devised a computational method that combines two different clustering methods to analyze CAGE expression data. The calculation procedure is as follows (Figure 1); firstly we decided on the number of clusters of TSS data (Figure 2), and then we used k-means clustering (non hierarchical). Secondly, we performed a tree view clustering (hierarchical clustering) to visualize the distance between the clusters (Figure 3, 4) by using the results from the first step. Our new method is useful in combining the merits of two calculation methods: the high degree of noise tolerance and calculation order of the non-hierarchical clustering and the improvement of the entire data by the second step.

Euclidean distance, one of the basic distance functions, was used as the distance metric of the first step, but it is possible to optimize the results by testing a variety of distance functions [14,16]. The use of other distance functions may give clusters with clearer GO term characteristics. Although other methods [24,25] have been proposed to reduce the calculation time of hierarchical clustering, these methods still require a huge amount of memory. In addition, they have a problem of making a visually understandable tree from such a large amount of data as ours. At this point, Tight Clustering is probably one of the powerful methods for a large amount of data [26]. One of the difficulties of this model-based method is the requirement of the choice of several parameters, which can cause some artifacts. In a widely used experimental method like microarray, the investigation of the parameter space can be done in advance. However, for a novel experimental method like CAGE, the a priori setting of parameters for a model based approach becomes very difficult. Here, a heuristic approach like ours might be the more appropriate choice.

The 70 clusters we obtained were well characterized by CAGE-TC expression patterns and gene ontology, proving our method's suitability for analyzing CAGE data. We have published another paper that shows the correlation between the expression pattern and upstream sequences of TSSs, noncoding RNA analysis, and alternative promoters in protein-coding genes [27]: if these data are combined, as in Figure 3 and 4, the features of gene expression regulation specific to biological function and upstream transcription regulatory elements can be easily and informatively described in a pleasing way. In Figure 3, we show that the clusters 5_(70) _and 69_(70)_, which belong to supergroup A_(70)_, were rich of noncoding RNAs located in the last exon. As mentioned in our previous article [27], noncoding RNA that are derived from 3'-UTR may function as regulatory RNA. The TCs in these clusters were accumulated in the visual cortex and in the embryo. This may suggest that the noncoding RNA derived from the last exon, which contains the 3'-UTR, may play particular roles in these tissues. However, we would not deeply discuss a biological meaning of the clusters in this paper because we do focus on the discussion concerning the methodology. Likewise, by using the method described here, we can gather new information on biological processes by combining and comparing different TSS-based data.

## Abbreviations

CAGE, cap analysis of gene expression; FANTOM, functional annotation of mouse; TU, transcription unit; TC, tag cluster; TSS, transcription start site; GO, gene ontology; nRSS, normalized residual sum of squares.

## Authors' contributions

KS and TK conceived the method of clustering. KS were responsible for the implementation of clustering software and drafted the manuscript. YO provided advice and conceived the biological validation sequence, and helped draft the manuscript. MCF collected the data. PC and YH provided advice and supervised the research group. All authors read and approved the final manuscript.

## Appendix

### Materials

All RNA samples used were the same as used in the FANTOM3 analysis [9]. Detailed information corresponding to RNA sample IDs used in this clustering can be seen in the FANTOM3 Basic Viewer [28]. We chose 127 libraries which has more than 1500 mapped tags from among 209 CAGE libraries. The CAGE libraries, which are non-normalized, unsubtracted, and unfractionated, were prepared according to Shiraki et al. [8]. 5'-End sequences of full-length transcripts (CAGE tags) were mapped to the mouse genome version UCSC mm5 by the procedure described by Carninci et al. in [9]. To establish the correspondence between CAGE tags and TUs, we used the Representative Transcript Set [29].

### Calculation of *P*-values of TC to GO association

TCs were easily connected to GO terms. Most TCs were included in specific TUs defined by the RTPS dataset [29], in which the TUs were connected to GO terms. *P*-values of the TC-GO association were calculated from the number of TCs connected to certain GO terms and the total number of TCs in the cluster by R Statistics software [30] in a one-sided Fisher's exact test. The term "Random" in Figure 3 and 4 shows whether each cluster significantly contains randomly chosen TCs. We did another test that randomized all the relations between TC and the expression. The Fisher's test yielded significant p-values in very few clusters. See supplementary web site [31].

### Non-coding RNA dataset

The non-coding RNAs were the FANTOM clones predicted to be non-coding by at least 2 out of 3 methods: CRITICA (Coding Region Identification Tool Invoking Comparative Analysis) [32], mTRANS and rsCDS [33].

### Software and expression data processing

The software used for the k-means (first step) was EISEN Cluster3 1.27 [34], maximum number of repetitions for the calculation of Figure 2, 3 and 4 were 5 and 100, respectively. Rand seed was 100 (for Figure 3, 4), and the tree view (second step) clustering procedure was Cluster3 1.31 [34], modified by our group. In this clustering, we used Euclidean distance (first step and second step: RNA sample clustering), and Uncentered correlation (second step: TC cluster clustering) for the distance metric. Pair-wise average-linkage was used in the hierarchical clustering algorithm. The expression values were converted to log-transformed tags per million. Programs (diff file) and some details, used in this paper, are available at the supplementary web site [31].
